# Biology and Management of High-Grade Myxofibrosarcoma: State of the Art and Future Perspectives

**DOI:** 10.3390/diagnostics13193022

**Published:** 2023-09-22

**Authors:** Jun Nishio, Shizuhide Nakayama

**Affiliations:** 1 Section of Orthopaedic Surgery, Department of Medicine, Fukuoka Dental College, 2-15-1 Tamura, Sawara-ku, Fukuoka 814-0193, Japan; 2 Department of Orthopaedic Surgery, Faculty of Medicine, Fukuoka University, 7-45-1 Nanakuma, Jonan-ku, Fukuoka 814-0180, Japan; n.shizuhide@gmail.com

**Keywords:** myxofibrosarcoma, MRI, PET, diagnosis, cytogenetics, pathogenesis, treatment

## Abstract

Myxofibrosarcoma (MFS) is one of the most common adult soft tissue sarcomas, typically arising in the extremities. Histologically, MFS is classified into three grades: low, intermediate, and high. Histological grades correlate with distant metastases and tumor-associated mortality. The diagnosis of MFS is challenging due to a lack of well-characterized immunohistochemical markers. High-grade MFS displays highly complex karyotypes with multiple copy number alterations. Recent integrated genomic studies have shown the predominance of somatic copy number aberrations. However, the molecular pathogenesis of high-grade MFS remains poorly understood. The standard treatment for localized MFS is surgical resection. The systemic treatment options for advanced disease are limited. This review provides an updated overview of the clinical and imaging features, pathogenesis, histopathology, and treatment of high-grade MFS.

## 1. Introduction

Myxoid soft tissue sarcomas (STSs) represent a heterogeneous group of mesenchymal neoplasms characterized by a predominantly myxoid stroma, including myxofibrosarcoma (MFS) [1]. MFS is a common type of STS that primarily arises in the extremities of elderly patients. It belongs to the fibroblastic/myofibroblastic tumor group according to the 2020 World Health Organization Classification of Soft Tissue Tumors [2]. The estimated incidence of MFS is less than 0.1 per 100,000 each year [3]. MFS is clinically characterized by a high risk of local recurrence related to its infiltrative growth pattern. Currently, MFS has been subdivided into three grades based on the degree of cellularity, nuclear pleomorphism, and proliferative activity [2]. Compared to low-grade MFS, intermediate- and high-grade MFSs show highly complex karyotypes with multiple copy number alterations and have a worse prognosis due to their aggressive behavior. Wide resection is the standard treatment for localized MFS. The treatment options in advanced disease are limited. It is often difficult to carry out robust research/clinical trials in a rare condition like MFS. This review highlights the clinical, radiological, histological, cytogenetic, and molecular genetic features of high-grade MFS. In addition, we will discuss the available treatment methods with the potential candidates for future therapies and ongoing clinical trials.

## 2. Clinical Characteristics

MFS can occur at any age but has a peak incidence in the sixth to eighth decades of life, with a slight male predominance. It usually presents as a slow-growing, often painless mass in the extremities. The involvement of the retroperitoneum and abdominal cavity is extremely uncommon. The presenting symptoms are typically related to the location of origin. More than half of cases develop in the subcutaneous tissues, with the remainder involving the underlying fascia and skeletal muscle [2]. Local recurrences occur in 10–61% of cases, irrespective of histological grade [4,5,6,7,8,9]. Notably, 19–50% of locally recurrent MFS cases progress to a higher histological grade with an attendant increase in metastatic potential [10,11,12]. Local recurrence within 12 months of initial surgery is associated with a higher mortality rate [13]. Low-grade MFS has no metastatic potential, whereas intermediate- and high-grade MFSs develop distant metastases in 16–38% of cases [4,6,14,15,16,17]. A recent epidemiological study from the Netherlands comprising 908 MFS patients indicates that median overall survival (OS) is 155 (range 0.1–215) months, with a 5-year OS of 67.7% [17]. The 5-year OS of MFS is better when compared to other types of STS [18]. It is currently recognized that age, sex, tumor size, histological grade, and surgical margins are the significant prognostic factors for MFS. Moreover, the presence of systemic inflammation has recently been reported to be associated with a worse prognosis in patients with high-grade MFS [19].

## 3. Imaging Features

### 3.1. Ultrasonography

Ultrasonography (US) is generally a first-line modality for the evaluation of palpable or visible superficial soft tissue nodules/masses. In addition, US is ideal in directing percutaneous biopsies. In US, MFS reveals a predominantly hypoechoic heterogeneous mass [20]. The presence of tail-like extensions along the fascial layer or ill-defined echogenic changes in the adjacent subcutaneous fat may be identified. However, echogenicity and vascularity can be variable for MFS based on the histological appearance.

### 3.2. MRI

**Figure 1:**
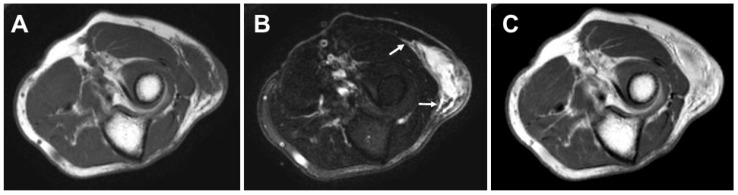
Axial magnetic resonance imaging of high-grade myxofibrosarcoma in the left elbow of a 71-year-old man. The lesion shows intermediate signal intensity on T1-weighted sequence (**A**) and very high signal intensity on T2-weighted spectral presaturation with inversion recovery (STIR) sequence (**B**). A tail sign (arrows) can be seen on STIR sequence. Contrast-enhanced T1-weighted sequence (**C**) demonstrates diffuse enhancement of the lesion.

Magnetic resonance imaging (MRI) is the preferred modality to evaluate soft tissue masses in routine sarcoma clinical practice. In addition, MRI is critical for optimal surgical planning. On MRI, the lesions are typically nodular or lobular in shape and display low to intermediate signal intensity on T1-weighted images and high signal intensity on T2-weighted images (Figure 1). Heterogeneity is often noted with all MR pulse sequences, particularly in high-grade MFS. An infiltrative growth pattern, referred to as a tail sign, can be seen on fluid-sensitive sequences [21,22,23,24]. The tail sign is not only valuable for suggesting the diagnosis of MFS, but its recognition is also essential in preoperative planning. Moreover, the presence of a tail sign has been recognized as a prognostic predictor for MFS [22,23,24,25,26,27]. Hemorrhage and necrosis may be seen within higher-grade lesions. In our experience, subcutaneous lesions may reveal perilesional edema likely owing to the lack of a pseudocapsule. Recently, Mühlhofer et al. reported that perilesional diffuse edema was significantly correlated with a poor OS [28]. Contrast-enhanced MRI demonstrates nodular and peripheral enhancement of the solid components. Diffuse enhancement may also be present in higher-grade lesions [29]. We believe that the use of contrast is essential to distinguish the tail sign from edema.

### 3.3. F-FDG PET/CT

Positron emission tomography/computed tomography (PET/CT) is not considered a standard imaging for staging and restaging STS, but it may exhibit a higher sensitivity in detecting distant metastases compared to conventional imaging in the initial stages [30]. In addition, PET/CT can be helpful in guiding the most aggressive area for biopsy in STS [31]. It is recognized that STSs with a myxoid component would show lower fluorodeoxyglucose (FDG) uptake than those without [32,33]. In our experience, high-grade MFS tends to have higher rates of FDG uptake than those of low-grade MFS. Recently, Macpherson et al. reported that the advantages of PET/CT were manifested during follow-up restaging and treatment response assessment in most cases of MFS [34].

## 4. Pathogenesis

**Figure 2:**
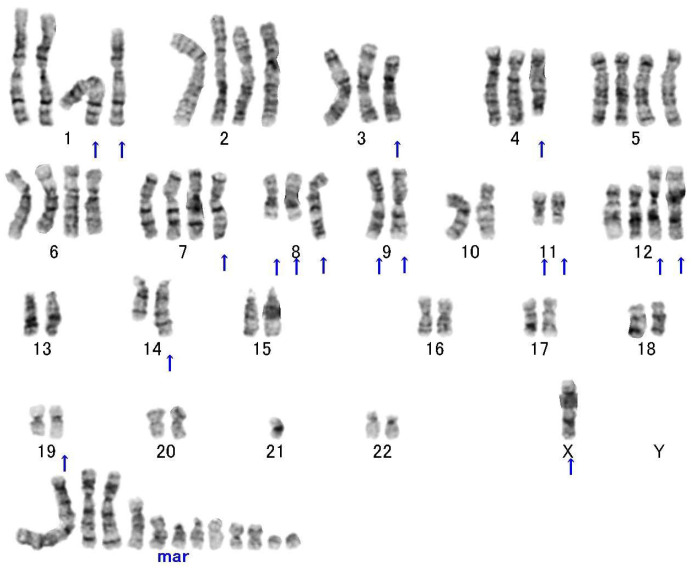
A representative G-banded karyotype of high-grade myxofibrosarcoma showing fairly complex chromosomal aberrations. Arrows indicate the structural chromosomal aberrations.

High-grade MFS is associated with very complex karyotypes lacking specific chromosomal aberrations [1,2,11] (Figure 2). Triploid and tetraploid ranges are noted in the majority of cases. MFS shares many chromosomal aberrations with undifferentiated pleomorphic sarcoma (UPS) [35]. The progression in grade is accompanied by an increase in cytogenetic alterations [11].

A conventional comparative genomic hybridization (CGH) study reveals gains of 19p and 19q, losses of 1q, 2q, 3p, 4q, 10q, 11q, and 13q, and high-level amplifications of the central region of chromosome 1, 5p, and 20q [36]. Interestingly, a gain of 5p and loss of 4q are not observed in low-grade MFS as opposed to higher-grade neoplasms. In addition, array CGH studies show gains of 7p21-22, 7q21-22, 7q31–35, 9q22, 12p13-pter, 12q15–21, 17q22–23, Xp11, and Xq12 and losses of 7p12, 7q11, 10p13–14, 10q25, 11p11–14, 11q23–25, 13q14–34, 20p11–12, and 21q22 [37,38]. Lee et al. reported that MET was expressed in 67% of primary localized MFS cases and its overexpression was highly related to deep location, higher grade, and more advanced stage [38]. Ma et al. also reported that MET overexpression was observed in 46.7% of cases with s correlation with higher grade and suggested that chromosome 7 polysomy, rather than the MET proto-oncogene, receptor tyrosine kinase (*MET*) amplification, might lead to the overexpression of the MET protein in MFS [39]. Patients with MET overexpression or chromosome 7 polysomy had a high risk of local recurrence and distant metastasis. Moreover, recent FoundationOne^®^ Heme testing demonstrated the presence of an upregulation of hepatocyte growth factor (*HGF*)*/MET* signaling in a subset of MFSs [40]. Based on these findings, we speculate that MET inhibitors may be an effective therapeutic option for high-grade MFS patients.

In general, high-grade MFS shows a higher amount of somatic copy number alterations than low-grade MFS [41]. Recent integrated genomic studies reveal ubiquitous genetic complexity in MFS, including the common occurrence of chromothripsis accompanied by local hypermutation [42,43,44]. These studies identified recurrently mutated/copy number altered genes such as tumor protein p53 (*TP53*), RB transcriptional corepressor 1 (*RB1*), cyclin dependent kinase inhibitor 2A (*CDKN2A*), cyclin dependent kinase inhibitor 2B (*CDKN2B*), neurofibromin 1 (*NF1*), neurotrophic receptor tyrosine kinase 1 (*NTRK1*), MDM2 proto-oncogene (*MDM2*), phosphatase and tensin homolog (*PTEN*), GNAS complex locus (*GNAS*), ATRX chromatin remodeler (*ATRX*), KRAS proto-oncogene, GTPase (*KRAS*), cyclin D1 (*CCND1*), Janus kinase 1 (*JAK1*), high density lipoprotein binding protein (*HDLBP*), mucin 17, cell surface associated (*MUC17*), filaggrin (*FLG*), and zinc finger protein 780A (*ZNF780A*). *TP53* mutation/loss, *CDKN2A*/*CDKN2B* loss, and *RB1* loss were the most frequent alterations in MFS. Interestingly, Yamashita et al. reported that *TP53* mutation/loss and *RB1* loss were significantly more frequently observed in high-grade than low-grade MFS and *RB1* loss was found to be a prognostic factor for adverse recurrence-free survival [45]. In addition, cyclin dependent kinase 6 (*CDK6*) amplification and its overexpression were found in 23.6% and 27.2% of cases, respectively [46]. CDK6 overexpression was associated with a worse outcome. Moreover, Ogura et al. detected a novel solute carrier family 37 member 3 (*SLC37A3*)-B-Raf proto-oncogene, serine/threonine kinase (*BRAF*) fusion gene in a single case [43].

In 2016, Okada et al. analyzed the gene expression profiles of 64 primary untreated high-grade MFSs and found that integrin subunit alpha 10 (*ITGA10*) expression was most significantly associated with disease-specific death and distant metastasis [47]. The authors also found that ITGA10 acts in association with trio Rho guanine nucleotide exchange factor (TRIO) and RPTOR independent companion of MTOR complex 2 (RICTOR), which are co-amplified on 5p and overexpressed in 42% of high-grade MFSs. Similarly, Heitzer et al. detected a co-amplification of *TRIO* and *RICTOR* in 44% of high-grade MFSs [41]. The authors also found that the overrepresentation of *RICTOR* alone was observed in only low-grade MFS and suggested that *TRIO* amplification might be a late genetic event. These findings demonstrate the importance of ITGA10/TRIO/RICTOR signaling for driving MFS progression and provide a novel potential treatment strategy for high-grade MFS patients.

In 2018, Lewin et al. investigated targetable genetic alterations in 43 MFS cases and 18 UPS cases using next-generation sequencing (NGS) [48]. The most commonly mutated gene was *TP53*. In addition, a solitary mutation in phosphatidylinositol-4,5-bisphosphate 3-kinase catalytic subunit alpha (*PIK3CA*) was detected in a single patient with MFS. *TP53* mutations were identified in 30% of patients with MFS and in 22% of patients with UPS. Currently, the role of NGS in differentiating high-grade MFS from UPS remains undefined.

Chromosome 5p is the most common copy number gain/amplification and includes the genes such as S-phase kinase associated protein 2 (*SKP2*) and alpha-methylacyl-CoA racemase (*AMACR*), in addition to *TRIO* and *RICTOR*. *SKR2* amplification was found in 38% of cases and associated with SKP2 immunohistochemical expression, adverse prognosticators, and worse patient survival [49]. *AMACR* amplification was found in 21% of cases through fluorescence in situ hybridization and associated with AMACR immunohistochemical expression and adverse prognosis [50]. Additionally, the overexpression of Ezrin (49%) and CD109 (10%) have been reported as potential biomarkers for the aggressive behavior of MFS [51,52]. Moreover, Conley et al. reported that MAGE family member A3 (MAGE-A3) was overexpressed in 41% of MFS/UPS and its overexpression was associated with a worse OS [53].

Interestingly, an online interactive tool, named Online consensus Survival analysis for Myxofibrosarcoma (OSmfs), has been developed to evaluate the prognostic value of certain genes in MFS [54]. This online analysis concludes that the overexpression of *ITGA10*, *CD109*, *CDK6*, *CDKN2A*, *MET*, *CCND1*, and Ezrin (*EZR*) predicts adverse survival for MFS patients. In addition, OSmfs suggests that the overexpression of lysophospholipase 1 (*LYPLA1*), DBF4 zinc finger B (*DBF4B*), matrix metallopeptidase 13 (*MMP13*), polo like kinase 1 (*PLK1*), transmembrane protein 158 (*TMEM158*), Wnt family member 5B (*WNT5B*), and RUNX family transcription factor 2 (*RUNX2*) may potentially predict a poor OS in MFS.

## 5. Histopathology

**Figure 3:**
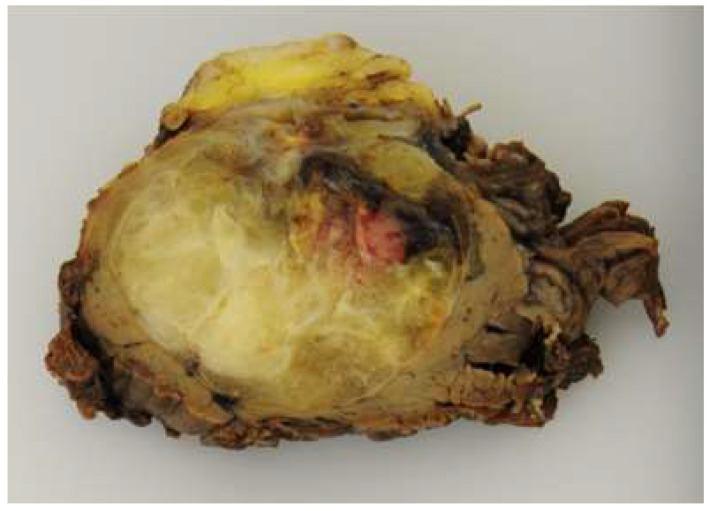
Overall appearance of high-grade myxofibrosarcoma. The tumor shows a multinodular growth pattern and a gelatinous, myxoid cut surface. Hemorrhage and necrosis can be seen.

On the whole, MFS usually appears as multiple nodules or an infiltrative single mass with a gelatinous, myxoid, and tan-white cut surface [2] (Figure 3). Hemorrhage and necrosis can be seen in high-grade MFS.

Histologically, MFS is classified into three grades based on the degree of cellularity, nuclear pleomorphism, and proliferative activity [2]. The elongated, curvilinear, thin-walled blood vessels and myxoid stroma (≥10%) are characteristic of MFS [8]. In addition, a rare epithelioid subtype of MFS has been described, with a poorer prognosis compared to conventional MFS [55]. Epithelioid MFS is composed predominantly of atypical epithelioid cells with abundant eosinophilic cytoplasm, round vesicular nuclei, and prominent nucleoli. The neoplastic cells are arranged in small clusters in the myxoid areas or forming sheets in the hypercellular areas [55]. Scoccianti et al. suggested that chemotherapy should be considered as an adjuvant treatment in this subtype [56].

**Figure 4:**
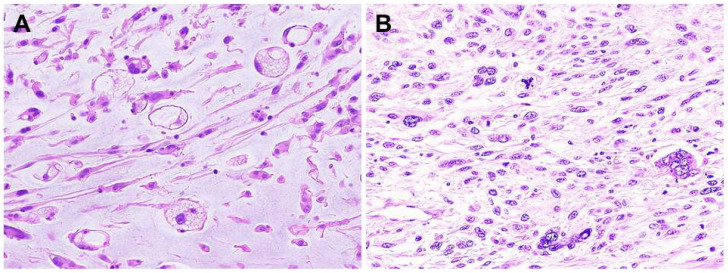
Histopathology of myxofibrosarcoma (MFS). (**A**) Low-grade MFS is composed of spindle cells with mildly atypical, hyperchromatic nuclei in a myxoid stroma. Pseudolipoblasts can be seen. (**B**) Bizarre, pleomorphic giant cells with eosinophilic cytoplasm are present in high-grade MFS, mimicking undifferentiated pleomorphic sarcoma.

Low-grade MFS consists of spindle cells with mildly atypical, hyperchromatic nuclei in a variably myxoid matrix (Figure 4A). Pseudolipoblasts containing cytoplasmic mucin may be seen [2]. Mitotic figures are rare and tumor necrosis is absent in low-grade MFS. In contrast, high-grade MFS is composed partly of solid sheets and fascicles of atypical spindle cells. Bizarre, pleomorphic giant cells are also present (Figure 4B). Mitotic figures often exceed 10 mitoses per 10 high-power fields. Atypical mitoses are common and tumor necrosis is variably present in high-grade MFS. At least focally, however, areas of a lower grade neoplasm with a prominent myxoid stroma are present [2]. Intermediate-grade MFS is more cellular and pleomorphic than purely low-grade MFS and often contains minute solid areas showing flank pleomorphism. However, intermediate-grade MFS lacks pronounced cellular pleomorphism and tumor necrosis [2]. The histological grades are summarized in Table 1.

Immunohistochemically, the neoplastic cells are occasionally positive for smooth muscle actin (SMA), muscle specific actin (MSA), and CD34 [2]. Immunostainings for S-100 protein and desmin are typically negative. Recent immunohistochemical studies demonstrate a strong expression of tumor endothelial marker 1 (TEM-1), also known as endosialin/CD248, in 88.2–100% of cases and suggest that TEM1 may be a suitable biomarker for fluorescence-guided surgery in MFS [57,58]. Most recently, we reported that glucose transporter 1 (GLUT-1) expression was seen in all MFS cases examined and suggested that GLUT-1 might be useful for the differential diagnosis of MFS and nodular fasciitis [59].

## 6. Management

### 6.1. Localized Disease

Wide resection is the standard treatment for local disease. In surgical practice, the selection of a procedure for an individual patient must be based on tumor size, location, stage, relationship with surrounding neurovascular and bone elements, and functional and cosmetic requirements. Deep intramuscular masses often require a composite reconstruction including muscle flap and skin graft. A resection with an R0 margin is more challenging for MFS due to its infiltrative growth [60]. Adequate margins must take into consideration both the resection margin width (quantity) and the type of anatomic barrier (quality) [61]. Fujiwara et al. concluded that a minimum resection margin of at least 1 cm should be the aim to minimize the risk of local recurrence [62]. Rhee et al. recommended a minimum of 2 cm margin width for the resection of MFS [63]. We now plan for margins of 2 cm from all of the enhancement areas of preoperative MRI. It should be kept in mind that the rate of local recurrence for MFS in margin-negative resection is relatively high compared to other STS subtypes [64]. Moreover, it must be considered that the epithelioid subtype is an unfavorable prognostic factor for local recurrence [56].

Radiation therapy (RT) can be used as neoadjuvant/adjuvant treatment strategies to improve local tumor control. Although the role of RT in the management of high-grade MFS is controversial, several retrospective studies have indicated that RT, in combination with surgery, is associated with a lower risk of local recurrence [14,65,66,67]. Adjuvant RT doses usually range from 50 to 70 gray depending on tumor size, location, and surgical margin status [65,66]. The possible drawbacks of RT include poor wound healing, pain, edema, fibrosis, and risk of secondary neoplasm. The long-term risk of radiation-induced sarcomas has not been reliably assessed in these studies. Mutter et al. suggested that clinical radioresistance might not be inherent to MFS of the extremities [65]. Look Hong et al. proposed that RT should be considered for all patients diagnosed with intermediate- or high-grade MFS [14]. The French National Group reported that a combination of R0 resection and adjuvant RT provided the best local tumor control [66]. On the other hand, Teurneau et al. indicated that there was no difference in the local recurrence rate depending on RT or not [68]. Recently, Kamio et al. reported that adjuvant RT did not contribute significantly to a better prognosis [69]. Further clinical trials are needed to better define the optimal treatment approaches for localized high-grade MFS.

### 6.2. Advanced Disease

The development of unresectable locally advanced or metastatic MFS is assocaited with a very poor prognosis. Accumulating more knowledge and experience is crucial in developing novel treatment strategies to combat advanced disease.

#### 6.2.1. Anthracycline-Based Therapy

Like other STS subtypes, anthracycline, with or without ifosfamide, is the first-line treatment for advanced MFS [70,71,72]. A randomized, controlled, phase 3 trial, comparing an anthracycline and ifosfamide combination (A + I) versus anthracycline alone, showed a significant improvement in progression-free survival (PFS) in the combination treatment group, but with no improvement in OS [71]. Moreover, a recent randomized open-label phase 3 trial suggested that A + I should remain the regimen to choose whenever neoadjuvant chemotherapy is used in patients with high-risk STS [73].

There is a retrospective case series concerning the role of anthracycline-based treatment in patients with advanced MFS [74]. In this case series, Colia et al. demonstrated that A + I was active in advanced MFS. The median PFS was 4 months and the median OS was 12 months. A partial response (PR) was observed in 4 (31%) of the 13 patients.

Most recently, Vanni et al. identified the down-regulation of several immunoglobulin genes and neutrophil-mediated immunity pathways in anthracycline-sensitive patients compared to anthracycline-resistant ones [75]. These results were reinforced by another recent study in which a high neutrophils-to-lymphocyte ratio was significantly associated with worse PFS in a case series of 99 STS patients, including MFS [76]. Indeed, it is known that neutrophils can remodel the extracellular matrix and promote angiogenesis, thereby stimulating tumor cell migration and metastasis. Moreover, neutrophils can suppress the cytolytic activity of lymphocytes [77,78].

#### 6.2.2. Gemcitabine-Based Therapy

Gemcitabine can be used as a monotherapy or in combination with docetaxel or dacarbazine in pretreated STS patients. A randomized phase 2 trial, comparing gemcitabine and docetaxel combination versus gemcitabine alone in metastatic STS, showed superiority in terms of PFS and OS with the combination [79]. The response to combination therapy was particularly notable in the UPS and leiomyosarcoma subgroups. Recently, Elkrief et al. reported that gemcitabine-based therapy was associated with encouraging response rates in metastatic MFS refractory to doxorubicin, similar to those observed in UPS [80]. The median PFS and OS were 8.5 months and 11.4 months, respectively. A radiological PR or complete response (CR) was observed in four (57%) of the seven patients. Although the exact role of gemcitabine remains unclear, gemcitabine-based therapy can be an effective option for metastatic MFS patients.

#### 6.2.3. Trabectedin

Trabectedin can be administered effectively and safely to patients with advanced STS at second- or later-line setting [81]. It has been approved by the United States Food and Drug Administration (FDA) for treatment of patients with unresectable or metastatic liposarcoma and leiomyosarcoma (L-sarcoma) who received a prior anthracycline-based regimen. Moreover, in 2015, trabectedin was approved in Japan for treatment of patients with STS after a clinical trial targeting translocation-related sarcoma [82]. In a preclinical study, trabectedin showed a cytotoxic activity in high-grade, patient-derived MFS primary cultures [83]. Preclinical data suggest that trabectedin may be effective for advanced MFS.

There are several retrospective studies concerning the role of trabectedin treatment in patients with advanced non-L-sarcomas, including MFS [84,85,86]. In the French RetrospectYon database analysis, of the 885 patients, 20 (2.3%) had MFS [84]. The median PFS was 2.8 months and the median OS was 8.1 months. In the Japanese musculoskeletal oncology group study, of the 140 patients, 3 (2.1%) had MFS [85]. The median PFS and OS were 5.3 and 10.6 months, respectively. In the Italian sarcoma group study, of the 512 patients, 13 (2.5%) had MFS [86]. The median PFS was 2.4 months and the median OS was 11.3 months in the non-L-sarcomas.

#### 6.2.4. Eribulin

Eribulin is currently licensed for use in patients with unresectable or metastatic liposarcoma who received a prior anthracycline-based regimen. Japan is the only country where eribulin is approved for all types of STS, including non-L-sarcomas such as MFS and UPS.

For retrospective studies, eribulin has demonstrated efficacy in Japanese patients with STS [87,88]. In the Japanese sarcoma group study, of the 256 patients, 5 (2.0%) had MFS [87]. The median OS was 11.1 months and an objective response (OR) was observed in one patient (20%). The authors concluded that the median OS of eribulin was similar to that of trabectedin. Another retrospective Japanese study of 82 STS patients included 45 patients with non-L-sarcomas [88]. The median PFS was 2.2 months and the median survival time was 7.9 months in the non-L-sarcomas. Patients with UPS had better OS than those with the other non-L-sarcomas. A PR was seen in one MFS patient. However, large-scale cohort studies are required to evaluate the clinical outcome of patients with advanced MFS after eribulin treatment.

#### 6.2.5. Pazopanib

Pazopanib is an oral multitargeted tyrosine kinase inhibitor (TKI) with anti-angiogenic and anti-tumorigenic properties and it has been approved in multiple countries as a second- or later-line treatment for patients with advanced non-adipocytic STS. A single-arm phase 2 trial (EORTC-62043) showed that pazopanib was inactive in the liposarcoma subgroup [89]. A subsequent randomized double-blind placebo-controlled phase 3 trial (NCT00753688) excluded liposarcomas on the basis of the EORTC-62043 data [90]. In the NCT00753688 trial for non-adipocytic STS, the median PFS was 4.6 months for pazopanib compared with 1.6 months for a placebo. There was no significant difference in median OS of 12.5 months for pazopanib compared with 10.7 months for the placebo. On the other hand, a prospective single-arm multicenter phase trial 2 (NCT01506596) was performed to support the efficacy of pazopanib of advanced liposarcoma [91]. In the NCT01506596 trial, the median PFS for patients with dedifferentiated liposarcoma was 6.24 months and the median OS among all patients was 12.6 months. Another phase 2 clinical trial (NCT01692496) also revealed that the median PFS was 3.5 months and the median OS was 16.4 months in the dedifferentiated liposarcoma subgroup [92]. These findings suggested that the use of pazopanib in treating advanced liposarcoma, especially dedifferentiated liposarcoma, may show promise [93].

Although limited by the small number of patients, there are several retrospective studies concerning the role of pazopanib treatment for advanced MFS [94,95]. A Japanese musculoskeletal oncology group study showed that, out of eight MFS patients, four (50%) had stable disease (SD), two (25%) had long SD, and two (25%) had progressive disease (PD) [94]. In the Indian retrospective study, Kataria et al. reported that, out of four MFS patients, two (50%) had SD and two (50%) had PD [95]. More recently, a prospective multicenter phase 2 trial (NCT02575066, acronym PASART-2) was performed to investigate the efficacy of neo-adjuvant pazopanib and concurrent external beam radiotherapy for high-risk, localized STS [96]. In the NCT02575066 trial, of the 25 patients, 8 (32%) had MFS. One (12.5%) had radiological PR and seven (87.5%) had SD. No pathological CR (≤5% viable cells) was observed in MFS. Further studies are needed to verify the efficacy of pazopanib in patients with advanced MFS.

Other TKIs, such as sunitinib [97], sorafenib [98], regorafenib [99], cediranib [100], apatinib [101], and anlotinib [102], have also been investigated in phase 2 trials in advanced STS. None are currently licensed for use in MFS.

#### 6.2.6. Immunotherapy

The major targets of FDA-approved immunotherapeutic antibodies are programmed cell death protein-1 (PD-1) and its ligand, programmed cell death ligand-1 (PD-L1) [103]. The prognostic value of PD-1/PD-L1 expression in STS remains controversial. Several studies have assessed the expression of PD-L1 in MFS [45,104,105,106,107,108]. The expression rate of PD-L1 has been reported to be approximately 0–35.6% in MFS. Yamashita et al. indicated that all PD-L1 positive cases were high-grade MFS [45]. Wunder et al. reported that high PD-L1 mRNA expression was not significantly associated with OS in 50 MFS patients [105]. In addition, the authors identified that the Th1 pathway was not activated in MFS. Smolle et al. showed that a higher prevalence of PD-L1, PD-1, and any tumor-infiltrating lymphocyte (TIL) phenotype was found in MFS compared with leiomyosarcoma and synovial sarcoma [106]. Likewise, *PD-L1* copy number gain was detected in 35% of the MFS cases [109].

Several studies have assessed the clinical benefit of immune checkpoint inhibitors (ICIs) as a monotherapy for STS [110,111,112,113]. In a single-arm open-label multicenter phase 2 trial (SARC028), pembrolizumab (anti-PD-1 antibody) provided an objective response rate (ORR) of 18% in the STS cohort [110]. The response was most noticeable in the UPS subtype (40% ORR). The results of SARC028 demonstrated promising activity in patients with UPS. In a retrospective study of the 88 metastatic STS patients, 47 (53.4%) received pembrolizumab monotherapy [111]. The ORR was 19.1% and a CR was seen in one UPS patient. In an open-label multicenter phase 2 trial to evaluate the efficacy of nivolumab (anti-PD-1 antibody) in 21 Japanese patients with advanced STS, including 2 MFS patients, the ORR was 0% and the median PFS was 1.4 months [112]. In a randomized open-label non-comparative multicenter phase 2 trial (Alliance A091401), 85 patients with metastatic sarcoma received either nivolumab alone or nivolumab in combination with ipilimumab (anti-cytotoxic T lymphocyte-associated antigen-4 (CTLA-4) antibody) [113]. The ORR was 5% in the nivolumab monotherapy group and 16% in the combination group. The median PFS was 1.7 and 4.1 months, respectively. The median OS was 10.7 and 14.3 months, respectively. These studies indicate that the clinical activity of single-agent ICI in STS is low and suggest that a dual checkpoint inhibitor may result in higher response rates.

There are several studies [114,115] and individual case reports [116,117,118,119,120,121,122] regarding the efficacy of ICIs for advanced MFS. In a retrospective study of the 61 advanced STS patients, 7 (11.5%) had MFS and received ICIs in combination with TKIs [114]. The ORR was 42.9% and all patients achieved SD. In a prospective clinical trial, PD-1 blockades demonstrated promising activity in patients with advanced MFS [115]. Among the five evaluable patients with MFS, two (40%) had CR/PR and two (40%) had SD. These results show that MFS as well as UPS are responsive to ICIs and suggest that MFS may have response rates that are near or above 40%.

Combinational therapies may ultimately prove more efficacious. A randomized open-label multicenter pivotal phase 2 trial (ENVASARC), to evaluate the effectiveness of envafolimab (anti-PD-L1 antibody) or envafolimab combined with ipilimumab in patients with refractory MFS and UFS, is currently ongoing. Another phase 2 study (NCT04332874) of concurrent systemic pembrolizumab and isolated limb infusion with melphalan and dactinomycin for patients with locally advanced or metastatic extremity sarcoma, including MFS, is also in progress [122]. Additionally, a randomized controlled phase 2 trial (SU2C-SARC032), to evaluate the safety and efficacy of neoadjuvant pembrolizumab with concurrent RT and adjuvant pembrolizumab compared to neoadjuvant RT alone in patients with high-risk extremity STS, including MFS, is currently under way [123]. The results from these trials shall be eagerly anticipated.

#### 6.2.7. Alternative Strategies

High-intensity focused ultrasound (HIFU) is a minimally invasive treatment modality that can ablate target tissue or tumors within the body. HIFU is usually guided, assessed, and monitored by either US (US-HIFU) or MRI (MR-HIFU). Recently, Zhao et al. reported on a patient with recurrent MFS who was treated with five cycles of low-power cumulative HIFU, without complications [124]. The patient has been disease free with a high quality of life for more than 30 months. Another minimally invasive alternative for the treatment of advanced or metastatic STSs is percutaneous image-guided cryotherapy [125,126,127]. This ablation technique can be used both as palliative treatment to reduce disease-related pain or as a curative treatment to achieve effective local tumor control [127]. However, more studies should be conducted to further evaluate the effectiveness of cryotherapy as a promising treatment for high-grade MFS.

## 7. Conclusions

MFS typically arises in the subcutaneous tissue of the extremities in older adults and has a high propensity for local recurrence. Distant metastases and tumor-related mortality are closely related to histological grades. Notably, epithelioid MFS behaves more aggressively. High-grade MFS displays highly complex karyotypes with multiple copy number alterations. At the transcriptomic level, high-grade MFS cannot be distinguished from UPS. Surgical resection is the mainstay of treatment for localized MFS, although the use of RT or systemic therapies in conjunction with surgery may be considered in selected patients. The management of advanced MFS is challenging. Novel therapeutic approaches using immune-oncology and molecular targeted agents may lead to a substantial improvement in the outcomes of patients with this devastating disease.

## Figures and Tables

**Table 1:** Histological grades of MFS.

Grade	Cellularity	Nuclear Pleomorphism	Mitotic Activity	Tumor Necrosis
low	low	rare	rare	absent
intermediate	moderate	mild/moderate	<10/10 HPF	absent
high	high	pronounced	≥10/10 HPF	present

HPF: high-power field.

## Data Availability

Not applicable.
